# Response of Healthcare Service Providers, to the Ebola Virus Disease Epidemic in the Democratic Republic of Congo’s North Kivu and Ituri Provinces

**DOI:** 10.29245/2578-3009/2023/S3.1107

**Published:** 2023-05-12

**Authors:** Tieman Diarra, Nkechi Onyeneho, Joseph Okeibunor, Amadou Baïlo DIALLO, Michel N’da Konan Yao, Mamoudou Harouna Djingarey, Ibrahima Socé FALL, Dick Chamla, Abdou Salam Gueye

**Affiliations:** 1 Independent Consultant, Mali; 2 University of Nigeria, Nsukka; 3 World Health Organization, Switzerland; 4 Independent Public Health Expert, Niger

**Keywords:** Health system, Healthcare providers, Epidemic response, Training, Protection, Health worker vulnerability, Health system vulnerability

## Abstract

Healthcare service providers are crucial for effective responses to disease outbreaks. However, their performance is dependent on the level of system inputs, people’s perception of the system, and their willingness to use health services. This study investigated the functionality of health services and healthcare providers in the Democratic Republic of Congo during the tenth Ebola virus disease outbreak. It employed qualitative methods, including 24 in-depth interviews of healthcare providers and community leaders, and 12 focus-group discussions with community members. The responses showed that the staff did not desert the health centers and remained at their jobs. Throughout this research, only one case of abandonment of duty by a nurse was reported. The healthcare system thus played a major role in responding to the COVID-19 pandemic. However, the healthcare service providers faced several challenges. Suggestions are made to enhance the contributions of healthcare service and its providers to health emergencies in the future.

## Introduction

The health system has been the backbone of the response to the Ebola Virus Disease (EVD) epidemic in the Democratic Republic of Congo (DRC)’s North Kivu and Ituri provinces. It involved all levels of the country’s health pyramid—health units, public and private health centers, referral health centers, referral general hospitals, and private clinics^1^—in both public and private service, as well as the modern healthcare system’s providers, who set up hand-washing amenities and a sorting system for patients and visitors. As healthcare workers had to deal with cases of infection despite being unqualified for responding to the epidemic, they ended up contracting EVD, and in some cases, dying from it. As with all epidemics, healthcare workers were most affected, and represented a significant part of the losses^2^.

Healthcare workers’ duties and working conditions exposed them to the disease^3^, and consequently, some died. Training helps to reduce the vulnerability of the health system and healthcare staff. Hence, health personnel received training to make them less vulnerable to the disease and be familiar with response activities. They were trained to protect themselves against the disease and ensure their responsibilities in the response. They were qualified to oversee suspected patients and use protective measures in their daily activities. They were also trained in epidemiological surveillance; infection prevention and control; raising awareness; and sensitizing community members. The health personnel benefited from capacity building, that enabled them to educate community health recruits, community relays, community leaders, and members of civil society organizations.

The epidemic also affected health facilities, which experienced a drop in attendance, owing to their being considered as places of transmission of the disease by the population, who feared them. This fear was reinforced by all kinds of rumors^2^ and resulted in a loss of income for health facilities, particularly the private, religious, and communal ones.

The free healthcare system also affected these facilities, which were confronted with other problems, such as the extent of their area of intervention, attitude of health workers who did not believe in the disease’s existence, community members’ negative perceptions fueled by rumors of the personnel, eventual lack of equipment, and attacks on health facilities^4–6^. Some units were destroyed because of rumors or events related to their response activities. Moreover, these facilities did not always have all the necessary equipment, or it was insufficient. In some cases, they even faced a lack of water.

Other problems encountered by the healthcare staff included the lack of standard operating procedures (SOPs) for some of the activities they were assigned. They were not always prepared for answering all the questions posed by the people during information sharing, sensitizations, or training activities. The national response authorities, like the zone coordinating doctors, highlighted the problem of anchoring the response in the health system. For them, while the response relied on both the commissions set up as pillars of the main interventions and the staff (of the health centers or health facilities), the responsibilities of the staff of the health facilities were seen to be limited in the emergency response. Overall, the involvement of the health system was real, and the participation of health providers was fundamental. The latter kept their jobs and performed activities that were under their responsibility. The health system and its providers played an important role in responding to the epidemic in the North Kivu and Ituri provinces.

This study first discusses the health system’s role in the response and its preparation for the response. It then discusses the impact of the epidemic on the activities of health facilities, and the strengths and weaknesses of the health system in the response. Finally, it addresses the issue of anchoring the response in the health system, difficulties of the health system in coping with the epidemic, and perspectives of health providers in improving the response.

## Study Design and Methods

### Study Design and Sites

This study was performed in DRC’s North Kivu and Ituri provinces, where the tenth EVD outbreak occurred. It explored and documented experiences and lessons around the response to the tenth EVD outbreak by adopting qualitative techniques of data collection. The methods of data gathering included in-depth interviews (IDIs) and focus group discussions (FGDs). This type of study requires a strong focus on individual actors rather than state actors.

Ituri is one of the 26 provinces of the DRC and its capital is the city of Bunia. The province is located to the northeast of the Ituri River and on the western side of Lake Albert. Ituri is a region of high plateaus (2000–5000 meters) with a large tropical forest and savannah landscape. The district has rare fauna, including the okapi—national animal of the Congo. As for flora, an important species is Mangongo, whose leaves are used by the Mbuti to build their homes. The population is composed primarily of Alur, Hema, Lendu, Ngiti, Bira, and Ndo-Okebo, with figures differing as to which of these groups constitute the largest percentage of the province’s population. The Mbuti, a pygmy ethnic group, reside primarily in the Ituri forest near the Okapi Wildlife Reserve, although some Mbuti have been forced into urban areas by deforestation, over-hunting, and violence. The Kilo-Moto gold mines are partly located in Ituri. In the beginning of the 21st century, petroleum reserves were found by Heritage Oil and Tullow Oil on the shores of Lake Albert.

North Kivu (French: *Nord-Kivu*) is a province bordering Lake Kivu in the eastern DRC. Its capital is Goma. North Kivu borders the provinces of Ituri to the north, Tshopo to the northwest, Maniema to the southwest, and South Kivu to the south. To the east, it borders the countries of Uganda and Rwanda. The province comprises three cities—Goma, Butembo and Beni—and six territories—Beni, Lubero, Masisi, Rutshuru, Nyiragongo, and Walikale. The province is home to the Virunga National Park, a World Heritage Site, containing the endangered mountain gorillas. Except for the heightened insecurity and isolation owing to rebel activities, North Kivu shares similar demographics with Ituri. The province is politically unstable, and since 1998 has been one of the flashpoints of the region’s military conflicts.

The 2018 or tenth Kivu EVD outbreak began on August 1, 2018, when four cases were confirmed as having tested positive for EVD in the eastern region of Kivu^7–9^. The Kivu outbreak included the Ituri Province, after the first case was confirmed on August 13^10^. This outbreak started just days after the end of the 2018 Équateur province’s DRC EVD outbreak^11,12^.

The affected province and general area are currently undergoing a military conflict, which is hindering treatment and prevention efforts. The World Health Organization’s (WHO’s) Deputy Director-General for Emergency Preparedness and Response has described the combination of military conflict and civilian distress as a potential “perfect storm,” that could lead to a rapid worsening of the outbreak. Owing to the deteriorating situation in North Kivu and surrounding areas, on September 27, 2018, the WHO raised the risk assessment at the national and regional level from “high” to “very high”.

### Study Population and Sampling

The study population comprised adults aged ≥ 18 years living in the community, as well as response team members. A 2010 estimate put North Kivu’s population at 5,767,945. Estimating the population at 70%, gave a population number of 4,614,356. With an annual growth rate of 3.2%, in 2019, North Kivu’s population was put at 7,658,406 and 5,360,884 for the general population and those aged ≥ 18 years, respectively. Contrastingly, a 2005 estimate put Ituri’s population at 4,037,561. An estimate of those aged ≥ 18 years at 70% population was 2,968,865. For 2019, the population was estimated at 6,275,305 and 4,392,714 for the general population and those aged ≥ 18 years, respectively.

The response team comprised over 10,000 persons, who were in different response pillars: surveillance, risk communication, social anthropology, and vaccination. Others included infection prevention and control, treatment and care, safe and dignified burial, as well as security, logistics, and administration, among others.

The center of the selected community was the reference point, where the team spun a pencil to determine the first route and first household, and thereafter moved to the right, to pick the next household, and continued until the number of households to be sampled was covered. Where there was a *cul-de-sac*, the step was retraced, and a turn was made to the left and then to the right to continue the sampling process.

A set of questions covering different thematic areas were developed to guide the discussions. The questions covered healthcare services in the community, EVD awareness and practices, and assessment of the different pillars of the response interventions.

For the FGDs, 8 to 12 persons were selected for each session. A minimum of two FGDs were conducted in the selected communities. There were separate FGD sessions for men and women in each of the communities. Overall, eight FGD sessions were conducted in each province.

In each of the communities, where FGD was performed, IDIs were conducted with their community or opinion leaders, and team leaders of the response pillars. Interviews were used to explore people’s opinions, views, and attitudes related to practices, for gaining insights into the outbreak and other socio-cultural factors that may have influenced attitudes toward their response to the outbreak. The FGD guide was used for the IDIs, focusing on the thematic areas of interest for the evaluation.

A structured questionnaire was used for collecting quantitative data from households. It addressed all the indicators that were used for answering the research questions, and was structured with results from the qualitative study. It was categorized into sections: socio-demographic data, perception of health problems in the community, knowledge of EVD, perceived epidemiology of EVD in the communities, and sources of information on EVD. Its questions also included issues on communication and community engagement, infection prevention and control in the communities, vaccination, surveillance, treatment and care, as well as covered, safe and dignified burial, psychosocial, logistic, and security issues.

All the interviews and discussions were tape-recorded, and detailed notes taken simultaneously, including verbal citations. Tape-recorded interviews were transcribed according to standard rules. Observations were also recorded, and together with discussion and interviews were triangulated with the quantitative data to arrive at conclusions.

### Data Management

Qualitative data consisting of FGDs and IDIs were transcribed from audio records to text. All textual data were analyzed using Atlas.ti software package (Scientific Development Software, Berlin, Germany). Data were analyzed according to themes corresponding to the indicators in the quantitative data and triangulated during presentation to enable complementary and analogous interpretation.

Given the continuous analytical process involved in qualitative analysis, the initial analysis of the key informant interviews and FGDs facilitated the final development of the structured questionnaire. This further enhanced triangulation between the two datasets to be collected. While the quantitative results provided statistical conclusions, the qualitative results placed emphasis on what was said, and provided illustrative quotes, that gave context and depth to the quantitative results.

### Ethical Considerations

The principle of do-no-harm was adhered to in this study. Approval for the study was acquired at the levels of the province, local administration, community, and household, and informed consent was verbally obtained from all individuals involved in this study. The WHO/AFRO Ethics Review Committee provided ethical approval (AFR/ERC/2018/09.3). All the researchers attended mandatory training, which included substantial discussions on ethical issues. Fifty percent of the research assistants were women, ensuring same-sex interviews and moderation of FGD sessions. The assistants were also trained and mandated to comply with child protection and gender-related sensitivity during the data collection process and visits.

## Results

### Health System’s Response

The health system has played and continues to play a large role in responding to the EVD outbreak. It involved both modern and traditional medicine providers, including modern health system providers, in both public and private service, and various health structures, such as public and private health centers, referral general hospitals, and private clinics.

Health services played an important role in the response to the EVD outbreak in the North Kivu and Ituri provinces. Healthcare workers were trained, and hand-washing amenities were put in place, along with a sorting system for patients and visitors. However, involvement varied according to the areas affected by the epidemic. This section reports on interviews conducted with heads of health facilities, directors of referral hospitals, health zone doctors, and different providers of health facilities.

The health personnel were trained in the context of the EVD outbreak. Additionally, capacity building was intended to prepare them to take appropriate action when facing suspected cases, as well as to protect themselves in their daily professional practice. In one health unit in Ngezi, two of the five health workers had been trained.

Health personnel were educated in Infection Prevention and Control. In some health facilities, staff benefited from several training sessions, designed not only to build their capacity to conduct awareness activities, but also to help them work within the context of the EVD epidemic by adopting prevention and protection measures. A nurse at a health center in Ngezi said: “*We have changed our ways of working. Our clothing has changed. We do waste segregation. We spontaneously wash our hands. We wear a fresh pair of gloves for each gesture. We also use a chlorine solution. The sorting is systematic for all those who come here.”*

Training is central to prepare health workers for the response.

The health personnel generally changed their conduct during their practices. A nurse from a health post in Ngezi said: *“Here, we have changed many things in our professional practice. For example, I was not taking any precautions while checking patients’ vital signs, but ever since Ebola, I take precautions. We disinfect the patients’ mattresses regularly. We don’t wait to do it until they are discharged, or before the bed is occupied by another patient. We wash the mosquito nets after the patients leave the hospital, which we didn’t do before.”* Wearing personal protective equipment (PPE) has now become customary in healthcare. A nurse at the Mandima Referral Hospital even told us of a change in the behavior of healthcare staff with the onset of the epidemic. She spoke of the observance of preventive measures by all health personnel in the hospital. For her, sorting patients became systematic. The arrangement of beds in the hospital ward was changed to maintain more distance.

The health centers were involved in the management of cases. Four suspected cases were processed through the Mandima Referral Hospital before being admitted to the Ebola Treatment Center (ETC) in Mambassa. The last person to be admitted was to be discharged from the ETC, the day after we interviewed a nurse from this hospital. The laboratory technician at Mandima Referral Hospital spoke of the changes in his daily work based on ICP training. He talked about protective measures, such as wearing gloves and specific gear, things that were not worn before the epidemic. He also talked about the staff’s involvement in monitoring, and their susceptibility in the workplace. He mentioned his own case: *“I was one of the people monitored. One day, I had a fever of 40 °C. I was sent to the ETC in Mambassa, where I stayed for three days. I tested negative for EVD, but was diagnosed as having malaria, for which I was treated. I was well taken care of.”*

Actual changes were made in health care facilities. A health worker spoke about the changes, that Rampera Hospital had implemented, such as implementation of ICP measures, including waste segregation and incineration. He told us of the measures adopted in the following terms: *“At the entrance of the hospital, every person goes through the sorting system and hand washing. Hence, when there is a case of fever, we can isolate the person, and then, call in the* response *team. However, none of the persons who had been isolated at our hospital, tested positive for EVD. We received the PPE before we had closer contact with patients. Now, we keep a certain distance between us and the patients, and wear gloves for every procedure. After our ICP training, we created a hygiene committee that gathers here (at the hospital), every Saturday.”*

### Impact of the Epidemic on the Activities of Health Facilities

Some health workers were involved in sensitization about the epidemic, and the means of preventing it. Community leaders, community relays, and religious people were sensitized so that they could inform other members of their communities. The head nurse of the Ngezi Health Center, in Bunia, said, *“We do a lot of sensitization. We go to the crossroads, cells, and places of prayer, where people gather, at a particular time. We have targeted 10 cells, and met religious leaders. At places of worship, we pass on messages about EVD and the epidemic, before and after the service. We are also working with the community relays. When there are suspected cases, we alert the people. We inform the staff involved in the* response, *and they come to check if it is a case of EVD or not. We are in contact with the teams that come to investigate. We also inform the* response *staff when there are deaths in the community.”*

In some health centers, there are workers involved in the follow up of contacts. Consequently, some health workers also contacted people outside of their work hours. The health staff raised awareness about the importance of vaccination. At the Mandima Referral General Hospital, one nurse oversaw the follow-up of 20 contacts, and other health workers had a similar number of contacts to follow up.

Some health workers were trained because of their involvement in different response teams, such as immunization and ICP activities. Some health personnel were involved in response activities and had major responsibilities, such as supervising implementation of the ICP activities. This was the case with a doctor at the Mandima Referral General Hospital.

The Rwampara health zone comprises 14 health areas, whose health facilities were involved in the response to EVD. However, according to a health worker, the construction of an isolation center for patients at the Rwampara Hospital led to its abandonment by the population. Most of the agents were vaccinated. At the Ngezi Health Center, only those who were not present on the day of the vaccination were not vaccinated.

### Health System: Strengths and Weaknesses

The health system was implemented in the North Kivu as well as Ituri provinces. It involved all Beni Health Zone’s facilities: its 18 health areas, Beni Referral General Hospital, and private facilities. However, the involvement of private facilities was mixed. Patients often went to such facilities in the hope of escaping the controls put in place by the public health services. The chief doctors of the health zones played an important role in the response, and even chaired certain commissions. For example, the Beni Zone’s Chief Medical Officer chaired the commission in charge of epidemiological surveillance.

While the health facilities played an important role in the response, they also showed weakness in the management of patients during the epidemic. For example, a health facility was unable to detect the first case with which it dealt. They admitted a woman, whom they treated for two days as a patient, who was sick from poisoning. According to an official of this health facility, although this patient did not show any signs of danger, she died two days later. Her body was handed over to her family, who took it home. As her family members were leaving for the burial, they were stopped by one of the units set up by the response team. When the body was checked, it tested positive for EVD. In the end, the body was buried, where it was tested. The funeral rites that were to follow the body’s arrival could have been a source of transmission of the EVD among the deceased’s family since it had not been detected by the structure that had taken care of the patient for two days. This situation reveals not only a weakness in the health structure, but also the strength of the system that was put in place as part of the response to EVD. Among communities, one of the strengths was this testing scheme at checkpoints that included measuring passengers’ temperature, handwashing, and disinfection of shoes. The staff at the checkpoints were very vigilant, and they could detect positive cases.

During the two days, when the health personnel had dealt with the patient, they had not taken any protective measures. According to this center’s administrative manager, *“The health staff had not detected any worrying signs and had not taken any precautions. Once, we got the information, everyone was vaccinated.”* When the second case came in, the health center isolated the patient who had tested positive for EVD, and the staff took the necessary precautions to avoid contamination. Afterward, the whole staff of the facility received training. They also established a sorting system, which was challenged by part of the neighborhood’s population.

All the healthcare workers or health center managers, whom we interviewed said, that the employees did not desert the health centers and continued their jobs. With the onset of the epidemic, they changed their ways of managing patients. During this study, only one case was reported of post abandonment by a registered nurse.

### Anchoring or Associating with the Health System in Response to the EVD Epidemic

It was not easy to connect the health system to the EVD response. According to Beni’s medical officer, communication was often missing. To him, it seemed as if the commissions were separate, and the staff at the health centers or facilities were considered as providers without any responsibilities. He said, “*Communication was difficult. Similarly, their telling me what to do, over and over again, was difficult, considering that I had already experienced the* EVD *epidemic. They tell you not to do this, but this and this. While the nurses involvement was low, there was involvement of the community relays who report to these nurses, and get paid for it. They are seen as members of commissions or working with commissions, despite being disconnected from the registered nurses, to whom they report, which is a source of frustration. Moreover, we often hear information on the radio, that ideally should have been shared first with the staff of the health facilities involved in the response*.”

A doctor in charge of the zone discussed the paradox of free healthcare, which did not lead to an increase in attendance at the Beni General Hospital. The proximity of the ETC to this health facility would have mitigated the expected effect of free healthcare. The infrastructure in the health zones did not always meet the demands of the response teams, who subsequently began to organize themselves to meet these requirements, particularly through the measures needed in the context of ICP. Health service personnel benefited from ICP training in the Beni Health Zone, even though not all agents received training. Health centers, along with community relays, largely contributed to eradicating the reluctance of community members to participate in the response activities. However, according to Beni Health Zone’s chief doctor, a problem exists with anchoring the response to the EVD response system, as he perceived the response as a parallel system, not integrated with the general implementation of activities in the health system. For him, the link with private structures was even more problematic.

A large part of the staff at health facilities were trained and vaccinated. In fact, at the Rampera Referral Hospital, except for one health worker, who was on leave at the time of training, the remaining 34 were trained, and all their agents were vaccinated. At the Nglogu Health Center, too, all the health workers were trained. One nurse had been trained in Shari, where she had been working before coming to Nglogu.

### Challenges Faced by the Health System in Reacting to the Epidemic

#### Several challenges existed, including the following

##### Decreased attendance at health facilities

The epidemic impacted the activities of health facilities everywhere, and led to a drop in attendance. A nurse at the Ngezi Health Center said, *“Before the epidemic, we had at least eight patients per day. Now, there are days when we only have one patient. Sometimes, there can be a week when we do not receive any patients. This has impacted our income. We now have difficulty meeting the costs of the health center, especially staff payments. Also, some patients are afraid of the PPE that we wear, and never come back after leaving the center. The face mask is the most frightening.”* The patients or their relatives began deserting the public health facilities thinking that they worked with the response teams. The head nurse of the Ngezi Health Center stated that patients go to private health facilities which hide information about their patients. He added: *“We work with the response teams. When we alert the* response *staff about a case, their agents come with an ambulance. People see all this, and they are so afraid, that they do not come to our health center anymore. Moreover, private health units hide information about their patients, and by doing so, they put the whole country in danger.”*

A nurse from a private health facility in the same town said, that the number of people coming for consultation had decreased. According to her, on some days the center received only two patients. At the time of this study, the maximum was 20 patients per day. She also stated that the health facility had many more people coming for consultation. This situation had an impact on the public health centers, and a nurse claimed that these centers had fewer resources. The laboratory technician at Mandima Referral General Hospital said there were days when the hospital had no patients. He added, *“People have stopped coming to this hospital because it had EVD cases, which have now been shifted to the ETC. Moreover, free care had not been established everywhere including at this hospital. Therefore, people go more often to health centers where free care has been established. They go to Mambassa because of free healthcare.”* A nurse at Mandima General Hospital emphasized that this situation affected the health facility’s revenues, and according to her, facing recurrent expenses would be a struggle. The decrease in attendance was a difficulty that was common to all public and private health centers as well as hospitals where we interviewed the staff or heads of establishments.

##### Coverage of the intervention zones of health facilities

Another major difficulty encountered was coverage owing to the extent of the intervention zones of health centers, which often had fewer staff. The lack of a means to travel was also mentioned. A doctor from the Mandima Referral Hospital spoke of the difficulties of traveling in the forest area, often up to 75 kilometers away. He stated that some trips took up to 8 hours. He mentioned the difficulties of traveling to Biakato, particularly the lack of means to travel, for the national party involved in the health zones and areas. When the community is not sufficiently prepared, their ability to cope with the epidemic becomes limited^2^. The extent of the health facilities’ intervention areas is also an obstacle to conducting sensitization activities among community members.

##### Multiplicity of messages

One of the problems mentioned is the multiplicity of messages coming from the partners, as stated by a nurse from the Mandima Referral General Hospital. For her, while the diversity of messages often led to confusion, the biggest challenge was resistance from the communities, which was an obstacle to the response. It compromised all actions at the community level and was a source of risk for the staff involved in the response, particularly the medical staff. She spoke of threats where machetes and stones were thrown at the vehicles of the EVD response teams. Collaboration with the response teams was also problematic, as it often led to attacks on health personnel by the population in the neighborhoods.

The main problems were as follows:

- **Multiplicity of questions asked by the population**. A laboratory technician at Mandina Referral General Hospital observed that training people involved in sensitization did not allow them to answer all the questions asked by the community members.
- **Attitude of some health workers**. One of the difficulties was the fact that some health workers did not believe in the existence of the disease. A doctor narrated the case of a laboratory assistant at a health center in Butembo who said that he did not believe in the existence of EVD. He spoke about the case of a doctor (the contact of a suspected case) who had refused vaccination, “*He refused to go to the ETC or take the vaccine. Eventually, he died. He had also separated from his wife and children.”* He also spoke about cases of refusal of vaccination by a doctor in Beni and a hygienist in Mandima.
- **Population’s perceptions of health workers**. The perceptions that some members of the community had of health workers was also a daily obstacle. According to a doctor: *“Health workers involved in the* response *are not well regarded. They say that we receive a lot of money.”*
- **SOPs**. The lack of SOPs for certain activities leads to different interpretations. The Chief Medical Officer of the Beni Health Zone deplored this situation because, according to him, those in charge were often divided on a topic as well as the course of action to be taken.
- **Multisectoral management**. Although the epidemic was being managed by the Ministry of Health and its partners, led by WHO, the multisectoral aspect of the response was not without problems, according to a doctor in charge of a health zone. For him, it was as if the response against the EVD was exclusively the Ministry of Health’s responsibility. According to him, all sectors should be involved to avoid the occurrence of an epidemic.
- **Lack of equipment**. Lack of equipment was a problem at the beginning, as declared by a health worker from Rwampara Hospital: *“At the beginning of the epidemic, we had difficulties working here. We did not have the appropriate equipment. We were in contact with the sick, but afterward, we got everything we needed for our work.”*
- **Withdrawal of partners**. Some health centers saw the departure of partners who had supported them before the detection of cases of patients who tested positive for EVD. For example, a health center in Bunia that had the support of 21 partners saw 15 of them leave.
- **Implementation of free healthcare**. All the health facilities did not benefit from free healthcare. This was the case with the general hospital in Bunia, which had to deal with a massive influx of refugees because of insecurity issues. The Mandima General Hospital also did not benefit from free care. Thus, every hospital involved in the response did not benefit from free care.
- **Attacks on health facilities**. The referral general hospital in Katwa, as well as its health personnel were attacked twice by armed groups. However, this situation did not lead to the abandoning of jobs by the 150 or more people who worked there. This hospital offered its pediatric ward to the response teams.
- **Lack of water**. Some health facilities were confronted with the problem of lack of water and electricity, such as the referral hospital in Katwa. However, this problem was not faced by the other health facilities, which we visited during this investigation.

### Health Professionals’ Perspectives

The EVD outbreak affected the professional practice of health workers. At the time of this study, health workers everywhere were wearing PPE, which they said they had not worn before. In many health facilities, they also wore gloves and boots. Before the epidemic, some health workers had been struck by the disease because of the non-observance of measures for their own protection and that of patients. In most cases, the health personnel were vaccinated and made proposals to improve the response.

Among other things, they proposed the following:

#### Greater involvement of local authorities in health activities in the fight against the EVD epidemic

This included rehabilitation of health centers to make them more functional, increased coverage of community-based surveillance, and greater integration of the grassroots community into community-based outreach and surveillance. A nurse said, *“As soon as community members see people coming from elsewhere, there is resistance from them. The more you involve the community, the better and the more successful you can be*.”

#### Strengthening the health system by providing means for the transport of patients

According to a doctor in charge of the area, it often took 5 hours for an ambulance, and this wait contributed to the population’s resistance.

#### Strengthening community involvement

This was mentioned by a doctor in charge of Beni area’s community watch. An example of greater involvement of the community was listening to them to consider the aspects they complain about, which are the cause of violent actions against the teams involved in the response.

#### Reinforcement of equipment at the health facilities

Notably, this comprised: hand-washing amenities, toilets, and water supply systems. The problem of social infrastructure was also highlighted, which included water and improving the lighting system.

#### Continuing education for health personnel

According to a doctor, the response teams should consist of trained people who can communicate with the communities and explain everything about EVD and its prevention.

#### Support for health personnel in the situation of loss of income in health facilities

The advent of the epidemic led to a global drop in attendance, and consequently, a decrease in the income of health facilities, as revealed through the interviews.

#### Integrating the EVD response into the health system, adopting a health zone approach, and integration into the health system

A general hospital’s director said, “*The* response *has not respected the health system. It should be based on the health zones, which have several public, private, and religious health centers and hospitals. All the health centers are involved in the response, which is an asset.”* The health system played a large role in the response. However, different pillars contributed to the success of the EVD outbreak response in both the provinces that were investigated.

## Discussion and Conclusion

Health services played an important role in the response to the EVD outbreak in the North Kivu and Ituri provinces. The health system was the backbone of the response. Various health structures were involved: health units, public and private health centers, referral health centers, referral general hospitals, and private clinics. Hospitals hosted ETCs. Modern healthcare providers from both the public and private sectors were present everywhere. They established hand-washing amenities and a system for sorting patients and visitors.

However, the health system was not prepared for the response as health workers were trained only after the outbreak. Some health workers had to manage patients when they were not prepared. Consequently, they contracted EVD, and some died. The training helped to reduce the vulnerability of the health system and its workers. Health workers were exposed by their work and its conditions. The inadequate use of PPE by the staff contributed to the development of the disease in both public and private health facilities.

The weaknesses of the health system were the breeding ground for the epidemic. The health system was not always robust enough to react to the challenges posed by the EVD epidemic in the DRC. Moreover, the methods used by the health personnel created fear among community members, as they were unfamiliar with them^13^. The national health system was not the only one that faced challenges, but globally too, the health system appeared to be fragmented without a strong structure^13^. Countries facing the EVD epidemic in West Africa were among those with a fragile health system and a deficit of healthcare personnel. The distribution of available staff was uneven between urban and rural areas^13^. This was also the reality in the DRC during the epidemic. It exposed the weaknesses of the health system, which was a point of vulnerability during the EVD epidemic.

Health personnel were trained to perform their responsibilities in the response. In all the health facilities that we visited the staff had been trained. This training was a great contribution to the response because it served to protect the health personnel themselves and enabled them to care for suspected patients under the required conditions. With training, the professional practice of health workers changed in the context of the epidemic. In the course of their work, they established all the protective measures. The health personnel’s training facilitated their involvement in response activities, such as epidemiological surveillance, prevention and control of infection, and also helped in informing and sensitizing community members as well as training community health recruits, community relays, community leaders, and members of civil society organizations.

This was the case in Uganda, where village volunteers were trained to conduct door-to-door outreach during an epidemic^14^. While it was noted elsewhere that the information given to health workers does not always translate into the knowledge used in their professional practice, this was not the case with the health professionals encountered in this study. Healthcare workers are often at risk. They are on the frontline during an epidemic. During the first EVD epidemic in Zaire (now DRC), infected health personnel died of the disease. Throughout the Kikwit epidemic in 1995, 30% of those infected were hospital healthcare staff^15^. The mortality rate of health personnel was 81%^5^. The vulnerability comes from inadequate information regarding the disease, namely on preventive measures, such as the adherence to safety procedures. During the seventh EVD outbreak in the DRC, among the 69 suspected, probable, or confirmed cases, 8 were health workers, who all died from the disease^5^. They had been infected after taking care of an index case—a pregnant woman. These individuals had taken care of the extraction of the deceased woman’s fetus, in accordance with the local traditions.

The epidemic led to a decline in the use of health facilities by the population. The health centers were deserted because they were perceived by community members as the places where the disease was transmitted and were thus seen as places of great risk. This was a problem insofar as some health facilities could no longer meet recurrent expenses, such as payments to staff, in the case of private religious or communal structures. The system of free healthcare also contributed to a drop in the income of health facilities, who were confronted with other problems, such as the extent of their intervention areas, attitudes of some health workers who did not believe in the existence of the disease, negative perceptions of health workers by members of the community owing to rumors about the disease and response, eventual lack of equipment, attacks against the health facilities, and eventual lack of water. Some health workers also complained about the lack of SOPs for certain activities they were responsible for implementing. For others, the multiplicity of messages did not facilitate their work among the population, who asked them all sorts of questions during information sharing, awareness-raising, or training activities.

There are traditions at funerals and burials that lead to exposure to the virus by allowing people to be in close contact with those who have died from the disease. This affected health workers during the seventh EVD outbreak in the DRC. In 2015, a number of health personnel engaged in the response to the EVD outbreak were infected or succumbed to the disease^16^. This was the case in Guinea, Liberia, and Sierra Leone. In the nine months of the epidemic, 318 health workers were infected, of which 48% died^16^. In Sierra Leone, more than half of the infected health workers died. In Liberia, the staff who came from abroad to support the EVD outbreak response were affected^5^.

The EVD outbreak affected the health system in Sierra Leone, with the risk of exposure to the disease. At the end of the epidemic, 221 of the 307 health workers who contracted the disease died^17^. Health workers paid a heavy price during the epidemic. During the 2014 epidemic in the DRC, all the eight infected healthcare workers died; in the West African epidemic, 57% of the infected health workers died^5^. Their work exposed them to the infection, especially when combined with the inadequate use of protective measures against the disease. Thus, the guidelines to deal with the obstacles of working in times of an epidemic were offered^18^.

The lack or inadequacy of PPE, lack of information on the transmission of the disease, and absence of compliance with prevention practices all exacerbated the toll on health workers. Across Africa, there was also a lack of medical personnel, particularly specialists. The fear of being infected also led to the desertion of health centers by some health workers. This fear was observed elsewhere^19^, including in Guinea^20^. The desertion of health services by workers also impacted the service supply. Therefore, the health system was not free of challenges while responding to the epidemic in the DRC.

The equipment provided to health services was not always sufficient, according to chief doctors of the zone. Health personnel were also at high risk through their professional practice. The knowledge acquired during training does not always translate into good practice^21^. This is partly owing to a lower perception of risk by some health professionals; this was the case among foreign healthcare staff, who came to work for the EVD response^22^.

In some cases, training does not lead to a substantial gain in knowledge for the health staff, as risk perception is not part of the training^23, 24^. Training is an integral part of response readinesss, both for health workers, and community members. Nonetheless, incorporating knowledge into professional practice is not always easy^25^. Several other factors also cause this decline in attendance at health services, such as lack of resources, recourse to traditional therapists, resorting to the church, poor quality of service, lack of medicines, and poor reception by providers^2^.

One of the problems highlighted by the national authorities in charge of the response, such as the coordinating doctors, was how the response was embedded in the health system. Thus, the response relied on both the commissions set up as pillars of the main interventions and the staff of the health centers or facilities. This situation was likely to reduce the responsibilities of health facility staff during emergency response.

A limitation of this study is its dependence on qualitative methods, which cannot be generalized. However, it provides some basic information, which helps to do a situation analysis of the health system in a restricted locality. This could form a baseline for the design of more robust studies using a mix-methods approach.

## Figures and Tables

**Table 1:** Distribution of participants in the in-depth interview (IDI) and focus group discussion (FGD) sessions by provinces.

Target	North Kivi				Ituri Province			
	Butembo		Beni		Mbuti		Bunia	
	IDI	FGD	IDI	FGD	IDI	FGD	IDI	FGD
Pillar leads	All		All		All		All	
Pillar members	2/pillar		2/pillar		2/pillar		2/pillar	
Community leaders^1^	≥2/ community		≥2/ community		≥2/ community		≥2/ community	
Leaders of survivor groups	≥2/ community		≥2/ community		≥2/ community		≥2/ community	
Community adult men		≥2 groups		≥2 groups		≥2 groups		≥2 groups
Community adult women		≥2 groups		≥2 groups		≥2 groups		≥2 groups
Community male youth		≥2 groups		≥2 groups		≥2 groups		≥2 groups
Community female youth		≥2 groups		≥2 groups		≥2 groups		≥2 groups
Survivors		≥2 groups		≥2 groups		≥2 groups		≥2 groups

1 Community leaders include: traditional, religious, political and opinion leaders

## Data Availability

The data that supports this study’s findings are not publicly available owing to their containing information that could compromise the privacy of the research participants. However, the data are available from the corresponding author (Joseph Okeibunor), upon reasonable request.
